# Who Cares If Not Supposed To? Moral Foundations, Consideration of Immediate Consequences, and Mask-Wearing Intentions After Revocation of the Legal Obligation To Do So

**DOI:** 10.5964/ejop.12957

**Published:** 2025-02-28

**Authors:** Iwona Nowakowska

**Affiliations:** 1 Institute of Psychology, Maria Grzegorzewska University, Warsaw, Poland; Dublin City University, Dublin, Ireland

**Keywords:** consideration of immediate consequences, COVID-19, mask-wearing, moral foundations, preventive measures

## Abstract

After two years of obligation to wear masks during the pandemic, in March 2022, the legal requirement was revoked for public spaces (except for medical facilities) in Poland. The aim of the study was to find out how individualizing moral foundations (focused on avoiding harm to others and concern for justice) shaped the intention to wear masks despite the revocation and how binding moral foundations (concentrated on respect for authorities, loyalty to the ingroup, and purity) and consideration of immediate consequences modify this relationship. For exploratory purposes, the same model was also tested for a retrospective declaration about the refusal to wear protective masks when legally required to do so. In both models, gender was controlled. *N* = 557 people from the general population participated in the online survey. Results showed that in the case of intention to wear masks after the obligation was revoked, individualizing moral foundations and female gender were positive predictors.

The lower the binding moral foundations and consideration of immediate consequences, the higher the effect of individualizing moral foundations. No interaction effects were found for a retrospective declaration of participants refusing to wear masks during the pandemic. However, positive predictors were male gender, binding moral foundations, and consideration of immediate consequences, whereas individualizing moral foundations were a negative predictor. The results suggest morality plays a role in forming health-related communication. Men should be targeted in order to enhance their acceptance of preventive measures.

The COVID-19 pandemic was an unprecedented event in many people’s lives and caused a worldwide public health crisis. It resulted in the introduction of various legal obligations, including social distancing and mask-wearing, aimed at caring for oneself and others to prevent the spread of the disease (Thunstrӧm et al., 2020). However, not everyone complied with these obligations, arguing that it compromised their liberty (Gostin & Hodge, 2020) or even denying the existence of the pandemic (Kaźmierczak et al., 2022).

One of the legal mandates was to wear protective masks in public (Howard et al., 2021). In Poland, on March 28^th^, 2022, the legal obligation to wear protective masks in public, closed spaces due to COVID-19 was revoked (the legal obligation to wear them in public, open spaces was revoked earlier by the Ministry of Health of Poland on Twitter, March 24^th^, 2022). It was argued that the number of infected people had decreased, and the wave of refugees from Ukraine did not change the pandemic situation.

The aim of the current study, conducted in the unique period between March 31^st^, 2022, and April 5^th^, 2022 (a few days after the obligation was revoked), was to test the potential predictors of mask-wearing intentions after the prevention law ended. Based on the Moral Foundations Theory (Graham et al., 2009) and the Norm Activation Model (Schwartz, 1970), we take into account the moral foundations (individualizing versus binding) endorsed by participants and the extent to which they consider immediate consequences (caring for the short-term gain of resigning from mask-wearing). An additional exploratory model regarding the refusal to wear masks before the revocation was tested to show whether similar patterns shape previous behaviors and future intentions.

The study aims to discuss the role of moral foundations in health behaviors. It can also assist in communicating prevention measures based on morality and consequence concerns, which could be helpful in the case of new pandemics or other public health threats.

## Mask-Wearing Effectiveness/Ineffectiveness as a COVID-19 Prevention Measure

Mask-wearing and other preventive measures have been introduced in many countries, including Poland, to lower the risk of spreading COVID-19 (Nowakowska & Zarazińska, 2023). A systematic review that considered observational and interventional studies conducted during the first wave of the pandemic suggested that mask-wearing effectively controlled the pandemic's spread (Mendez-Brito et al., 2021). Similarly, a systematic review and meta-analysis of observational and interventional data from 2020–2021 indicated that mask-wearing, handwashing, and physical distancing were associated with reducing COVID-19 cases (Talic et al., 2021).

A further systematic review of studies from 2020–2023 (Boulos et al., 2023) suggested that more research found that mask-wearing reduced infection rates rather than had no effect or favored control groups However, this conclusion is not definitive, and the studies were conducted before the highly transmissible Omicron variant emerged. There is a clear need for further research to fully understand the effectiveness of mask-wearing, especially in the context of new variants. Additionally, 95% of the reviewed studies were at critical risk of bias in at least one domain, often failing to distinguish between mask-wearing and concurrent interventions’ specific effects.

Finally, the most recent review of RCTs only (Jefferson et al., 2023) highlighted a contradiction in the evidence. It suggested that wearing masks in community settings made little or no difference in the case of influenza-like or COVID-19-like illnesses compared to not wearing masks. However, the harms of mask-wearing were rarely measured in the reviewed RCTs or were poorly reported. This evidence needs to be considered along with other available evidence. Nevertheless, the current study focuses on community members’ behaviors in light of governmental directions when the study was conducted. Generally, mask-wearing was considered a socially responsible behavior at the time of the pandemic (Chi et al., 2022; Kaul & Palmer, 2023). However, mask-wearing mandates induced polarized reactions, including social media encouragement to put masks on or take them off (Lang et al., 2021). Notably, the resistance among some members of society focused on how mask-wearing can compromise human rights and contribute to the consolidation of societal inequalities (Czarnecki & Skowrońska, 2022).

## Moral Foundations and Compliance With COVID-19 Prevention Measures

During the pandemic, it was found that moral foundations may serve as a framework for encouraging preventive behaviors, including mask-wearing (Chan, 2021; Nowakowska & Zarazińska, 2023). According to the Moral Foundations Theory (Graham et al., 2009; 2011; Waytz et al., 2019), people judge the appropriateness of behaviors and assess them as “right” or “wrong” based on intuition, not on conscious thought. Conscious processes only occur after.

The theory suggests five foundational moral domains, according to which people form their judgments intuitively (Graham et al., 2009). These five moral domains can be further grouped into individualizing (individual-centered), consisting of harm/care and fairness/reciprocity, and binding (group-centered), consisting of the three latter foundations (Graham et al., 2011; Haidt, 2012). These foundations are described as stable dispositions, despite the contextual factors (e.g., experimental manipulations) that can temporarily amend individuals’ moral foundations (Haidt et al., 2009). Table 1 presents an overview of the theory and a description of the moral foundations.

**Table 1:** Overview of the Moral Foundations Theory

Overarching moral domain	Moral domain description	Moral foundation forming the domain	Moral foundation description
Individualizing	Concerns what is best for individuals in society and what leads to equality and the well-being of other people. It promotes empathy and prosocial behaviors.	Harm/Care	Regards aversion to harm and promoting care for other people.
		Fairness/Reciprocity	Pertains to valuing fair behavior and reciprocal practices that promote equality.
Binding	Underlines the importance of tying together social groups by preserving roles and hierarchies.	Ingroup/Loyalty	Concerns sacrifice for own ingroup's benefits.
		Authority/Respect	Regards obedience to leaders, authority figures, hierarchy, and traditions and respect toward them.
		Purity/Sanctity	Highlights the importance of bodily purity and avoidance of what is contaminated and degrading.

*Note*. Source is own preparation based on Clark et al. (2017), Graham et al. (2009; 2011), and Milesi and Alberici (2018).

Individualizing-oriented people have a higher propensity to comply with COVID-19 preventive behaviors (Chan, 2021), assess the efficacy of COVID-19 preventive behaviors (including mask-wearing) more positively, and accept COVID-19 conspiracy theories to a lower degree (Nowakowska & Zarazińska, 2023). This is probably due to the empathic component of the harm/care foundation and the concern for fairness because if everyone complies, then the reciprocal interaction would also be to comply. Thus, it can be hypothesized that:

(**H1**) An individualizing orientation is positively related to mask-wearing intentions after the mask-wearing obligation is revoked.

A binding orientation, due to the concern for preserving the *status quo* (Day et al., 2014; Graham et al., 2009), which could, for example, manifest in not wearing extra accessories to prevent the spread of COVID-19, may be associated with less acceptance for mask wearing. Anti-mask views were positively related to binding moral foundations of authority/respect and purity/sanctity but negatively related to ingroup/loyalty (Mejova et al., 2023). Furthermore, binding-oriented people tended to assess the efficacy of COVID-19 preventive behaviors (including mask-wearing) more negatively and endorse COVID-19 conspiracy theories more (Nowakowska & Zarazińska, 2023). Thus, it can be hypothesized that:

(**H2**) A binding orientation is negatively linked to mask-wearing intentions after the mask-wearing obligation is revoked.

Individualizing and binding moral foundations are distinct. However, they are not mutually exclusive. Profile analyses show that some people tend to endorse all moral foundations at a similar level (Nowakowska & Zarazińska, 2023), and in some cases, an individual-protection-oriented-binding morality can arise, producing, for example, ostracization (Graham et al., 2020; Janoff-Bulman & Carnes, 2013). Given that the government (authority) announced that the legal obligation to wear masks would be revoked based on arguments of the diminishing threat to individuals and this was approved by the ingroup, a binding orientation could weaken the individualizing moral foundations’ relationship with mask-wearing intentions. It can therefore be hypothesized that:

(**H3**) The effect of individualizing moral foundations on mask-wearing intentions after the legal obligation to wear a mask is revoked is weaker the higher the binding moral foundations.

## Consideration of Immediate Consequences and Compliance With COVID-19 Prevention Measures

According to the Moral Foundations Theory, the moral judgment process appears at the first stage of deciding what behavior to undertake. The second stage is more conscious and based on reasoning. This is where some overlap with the Norm Activation Theory appears (Schwartz, 1970). In line with this, moral norms activate when a person sees the consequences of their behavior and assess them as manageable.

Consideration of immediate consequences (Strathman et al., 1994) describes to what extent an individual takes into account the immediate consequences of their own behavior. It is linked to lower self-control than consideration of distant (future) consequences (Joireman et al., 2008). Graham and colleagues (2020) found that low self-control is the strongest predictor of non-compliance with social distancing rules, which was higher for people who endorsed binding moral foundations. Nevertheless, it should be acknowledged that in this study, consideration of immediate consequences was not investigated directly. However, a study by Joireman and colleagues (2012) showed that people who consider immediate consequences are more inclined to engage in unhealthy behavior patterns (i.e., eating) because of the short-lived pleasure they might give.

Considering the immediate gains of “letting go” of the masks (e.g., personal comfort) may be negatively related to continuing to wear masks after the revocation of the obligation to do so. Taking into account this evidence, we hypothesize that:

(**H4**) Consideration of immediate consequences is negatively linked to mask-wearing intentions after the revocation of the law.

Similar to binding moral foundations, considering immediate consequences can serve as a “temptation” for individualizing-oriented people to not continue wearing masks, especially when they are not required to. It is possible that the reduced risk can activate the belief that there is no need to take care any longer, and mask-wearing is no longer a matter of fairness, as it is not expected. Moreover, reciprocity concerns should not be relevant anymore—if other people do not wear masks, then the symmetrical action is not to wear them. If the majority decides to follow the new rule of not wearing masks, then the immediate and negative consequence of continuing to do so could be a lack of understanding from others. Thus, it can be hypothesized that:

(**H5**) The effect of individualizing moral foundations on mask-wearing intentions after the legal obligation to wear a mask is revoked is weaker the higher the consideration of immediate consequences.

## Current Study

The aim of the current study, conducted in the unique period between March 31^st^, 2022, and April 5^th^, 2022 (a few days after the obligation to wear masks was revoked in Poland), is to test the potential predictors (individualizing moral foundations and their interactions with binding moral foundations and consideration of immediate consequences, as well as simple effects of binding moral foundations and consideration of immediate consequences) of mask-wearing intentions right after the prevention law ended. For exploratory purposes, in a second model, we check whether self-reported mask-wearing behaviors before the revocation of the law are predicted by the same individual differences (individualizing/binding moral foundations, consideration of immediate consequences, and interactions are similarly hypothesized as for mask-wearing after the revocation of the law).

For both models, gender will be controlled, as gender was consistently found to be related to mask-wearing behaviors during the pandemic (Chuang & Liu, 2020; Haischer et al., 2020; Looi, 2022). Men were less keen to comply with this preventive measure than women, which led to the coining of the term “toxic mask-ulinity” (Palmer & Peterson, 2020), which is a wordplay based on the “toxic masculinity” concept that describes the unhealthy practices of masculinity, matching negative stereotypes about the male gender. These practices might encompass aggressiveness, oppression of weaker people, and entitlement (Kupers, 2005; Waling, 2019). It was interpreted as a function of higher risk aversion in women (Chuang & Liu, 2020).

The study is unique in its timing (a few days after the obligation revocation) and is relevant to the fields of psychology and public health. The study was conducted at times when policymakers expected this behavior and considered it socially responsible. We focus on how people intend to behave when legal health-related regulations change. Understanding people’s behavioral intentions in the face of changing legal circumstances can help predict real-life behaviors when mask-wearing or other health-related legal obligations are revoked. Finding individual differences that correlate to such behavioral intentions is essential to observe who is most likely to sustain behaviors that were previously legally required and who is least likely to undertake the behavior regardless of the law or gives it up as soon as they are not obliged to perform it. The results may help form prevention-related messages and target specific moral concerns to promote socially responsible behaviors. Moreover, they contribute to the body of research on moral foundations and consideration of immediate consequences, adding to the knowledge of the links between morality and behavior and future discounting and behavior.

## Method

### Participants

A total of 557 people participated in the study, 400 women (71.8%), 145 men (26.0%), and 12 people of other gender or preferring not to disclose gender information (2.2%). The age range was between 18 and 71 years (*M* = 24.44; *SD* = 5.69). Eighty-one people (14.5%) lived in a village, 59 (10.6%) in a town with up to 50,000 inhabitants, 55 (9.9%) in a town with 50,000-100,000 inhabitants, 122 (21.9%) in a town with 100,000–500,000 inhabitants, and 240 (43.1%) in a town with over 500,000 inhabitants. Regarding the last finished level of education, one person (0.2%) had vocational education, 251 (45.1%) had high school education, 190 (34.1%) had a Bachelor's degree, 103 (18.5%) a Master's degree, 7 (1.3%) a PhD degree or higher scientific degree, and 5 (0.9%) other education status. For socioeconomic status rated from *1 – I can’t afford basic expenses*, to *10 – I can afford all necessary expenses and save money monthly*, participants ranged from 1–10 (*M* = 7.12; *SD* = 2.05). Two hundred and nine people (37.5%) stated having contracted/suffered from COVID-19 at least once in their lives, 164 (29.4%) declared that had not been the case, and 184 (33.0%) were unsure about it. Five hundred people (89.8%) indicated knowing at least one person in their close social circle who had gone through COVID-19, 26 (4.7%) responded having no such person in their close circle, and 31 (5.6%) were unsure about it.

### Procedure

On March 28^th^, 2022, the legal obligation of wearing protective masks in public spaces (except for medical facilities) was revoked. The study was performed between March 31^st^, 2022, and April 5^th^, 2022, to capture the beginning of the new reality period. The study was performed online, and the participants were recruited through Facebook using the snowball sampling technique; the information about the study was posted on various student and city groups across Poland to access people of various ages and places of residence. Initially, respondents were informed that the study aimed to assess current mask-wearing intentions. All participants consented to participate prior to starting the survey, and the study was anonymous without the consequences of giving it up. There was no remuneration for participating.

### Measures

#### Moral Foundations

Moral foundations were assessed with the Moral Foundations Questionnaire-MFQ (Graham et al., 2011) in a Polish adaptation (Jarmakowski-Kostrzanowski & Jarmakowska-Kostrzanowska, 2016). The tool consists of 30 items and is a self-report. It enables us to determine which of five foundational moral domains (harm/care, fairness/reciprocity, ingroup/loyalty, authority/respect, purity/sanctity) are essential for people’s decision-making. For the current study, the domains were further aggregated into individualizing and binding, as suggested by the literature. It consists of two parts, 15 items each. In the first one, the participants have to decide whether a particular issue is relevant to their moral decision-making, e.g., *Whether or not some people were treated differently from others*, rating their responses on a scale from 1 (not at all relevant) to 6 (extremely relevant). In the second part, the participants agree or disagree with moral statements, e.g., *People should not do things that are disgusting, even if no one is harmed,* rating their responses on a scale from 1 (*Strongly disagree*) to 6 (*Strongly agree*). Both parts of the questionnaire contain items that constitute the five subscales. Despite the different construction of questions, according to the authors of the scale, the items from both parts are taken together when calculating the scores (Graham et al., 2011). The scores were computed by calculating means for each subscale according to the key. Cronbach’s α in the current study for the subscales were: .75 for individualizing moral foundations and .87 for binding moral foundations.

#### Consideration of Immediate Consequences

This was assessed with the Consideration of Future Consequences Scale-14 (Joireman et al., 2012; Polish adaptation: Frydrysiak, 2018). It measures how much people consider future and immediate consequences in their decisions. The scale consists of two subscales: Consideration of Immediate Consequences and Consideration of Future Consequences, the first of which is important for the current analysis. A sample item from this subscale is: *My behavior is only influenced by the immediate (i.e., a matter of days or weeks) outcomes of my actions*. The answers are marked on a 7-point Likert scale (1 – *Not at all like me*; 7 – *Very much like me*). The general score was computed as the mean of the items relevant to the subscale. Cronbach’s α for the Consideration of Immediate Consequences scale was .75.

#### Mask-Wearing Refusal Before the Obligation Revocation

This was measured with one item of own construction: *Before the revocation of the obligation to wear protective masks, most often, I did not wear any*. The participants gave their answers on a scale ranging from 0 (*Totally disagree*) to 100 (*Totally agree*).

#### The Current Mask-Wearing Intention

This was measured using a 6-item survey of own construction. The instruction provided was: *On Monday, March 28^th^, 2022, the obligation for covering the mouth and nose in public spaces was revoked, with some exemptions (e.g., medical institutions). Read each of the following statements about your views carefully and mark on a scale from 0–100 to which extent you agree with them.* The participants gave their answers on a scale ranging from 0 (*Totally disagree*) to 100 (*Totally agree*). A sample item was *If someone asks me to wear a protective mask and it is not required in that place, I will wear one.*

Principal Component Analysis (PCA) was run to discover the structure of the survey. The criterion for choosing the number of factors was an Eigenvalue greater than 1. PCA suggested one factor accounting for 73.09% of the variance with all items with loadings over .79. K-M-O metric was .715, and Bartlett's sphericity test was statistically significant, χ^2^(15) = 3403.48; *p* < .001.

The general score was computed by calculating the items’ mean, acknowledging the two items’ reverse coding. The Cronbach’s α for this scale was .93. The results of the exploratory factor analysis, along with the complete list of items, are provided in the Appendix.

### Analytic Strategy

IBM SPSS 28.0.1.0 for Windows was used for data analysis. First, Pearson’s r correlations were used to explore the relationships between variables. Then, linear regression analysis with interaction was used to predict current mask-wearing intention. Next, simple slopes *post hoc* tests were performed using PROCESS 4.3 for SPSS macro (Hayes, 2018). All variables were standardized to *z*-scores prior to computing interaction variables and for *post hoc* interaction tests.

### Open Data

Open data, materials in original and English versions, and commands to recreate the analyses for the article can be found at Nowakowska (2024).

## Results

First, in Table 2, we present a correlation analysis along with descriptive statistics for the variables of interest to provide the first insight into the relationships between the variables.

**Table 2:** Pearson’s r Correlation Analysis and Descriptive Statistics

Variable	1	2	3	4	5	6
1. Gender (0 = female, 1 = male)	–					
2. Individualizing moral foundations	-.23***	–				
3. Binding moral foundations	.05	.11**	–			
4. Consideration of immediate consequences	.02	-.05	.01	–		
5. Mask-wearing refusal before the obligation revocation	.22***	-.24***	.20***	.09*	–	
6. Current mask-wearing intention	-.20***	.27***	-.18***	-.17***	-.58***	–
*M*	–	4.82	3.42	3.61	29.21	40.22
*SD*	–	.56	.76	.96	38.16	34.88

*Note.* Correlations with gender are provided for *N* = 545 (people who declared no information on gender are excluded).

*p < .05. **p < .01. ***p < .001.

Next, a regression with interaction analysis was performed to predict current mask-wearing intention. Durbin-Watson’s statistic for the whole model was 1.99. Table 3 shows the results of this analysis.

**Table 3:** Predicting Current Mask-Wearing Intentions: Regression Analysis Results

Predictor	*B*	*SE*	β	*t*	*p*	VIF
1. Gender (0 = female, 1 = male)	-11.05	3.23	-.14	-3.42	< .001	1.08
2. Individualizing moral foundations	8.55	1.47	.24	5.80	< .001	1.08
3. Binding moral foundations	-5.99	1.43	-.17	-4.18	< .001	1.06
4. Consideration of immediate consequences	-5.43	1.37	-.16	-3.96	< .001	1.01
5. Individualizing moral foundations * Binding moral foundations	-3.45	1.47	-.10	-2.36	.019	1.07
6. Individualizing moral foundations * Consideration of immediate consequences	-2.84	1.34	-.08	-2.12	.035	1.02
*F*(6; 538)	17.41					
*P*	< .001					
*R^2^_adj_*	.153					

Data from Table 3 shows that female gender and individualizing moral foundations were significant positive predictors of current mask-wearing intention. Negative and significant predictors were binding moral foundations, consideration of immediate consequences, the interaction between individualizing and binding moral foundations, and the interaction between individualizing moral foundations and consideration of immediate consequences.

**Figure 1:**
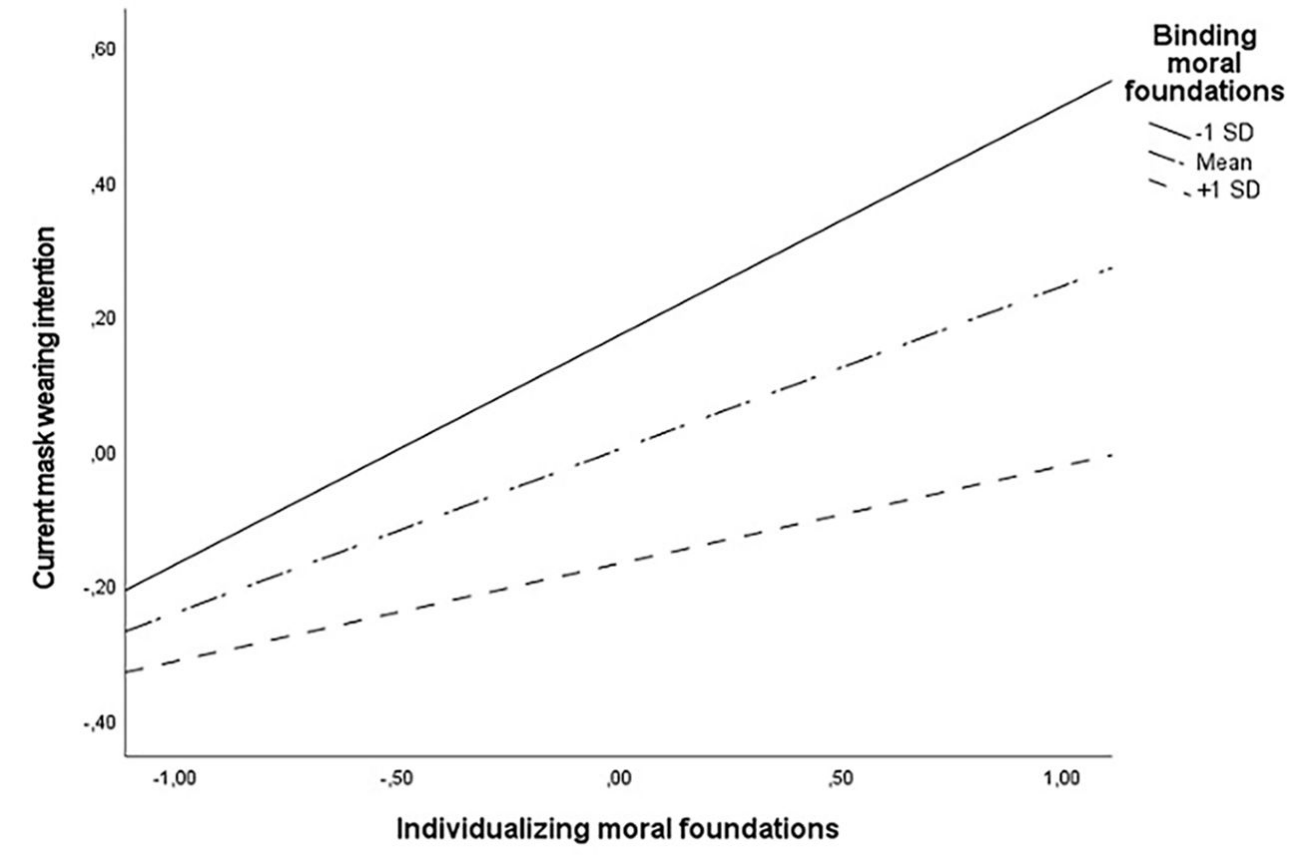
Interaction Between Individualizing and Binding Moral Foundations in Predicting Current Mask-Wearing Intention

Simple slopes *post hoc* tests indicated that individualizing moral foundations were related to current mask-wearing intentions on all levels of binding moral foundations (other variables and interaction from the regression analysis controlled). However, the relationship differed in strength. It was the strongest when the binding moral foundations were low, *B* = .34, *p* < .001; less strong when they were average, *B* = .24; *p* < .001, and the weakest when they were high, *B* = .15; *p* < .05. Figure 1 shows the visualization of this interaction.

**Figure 2:**
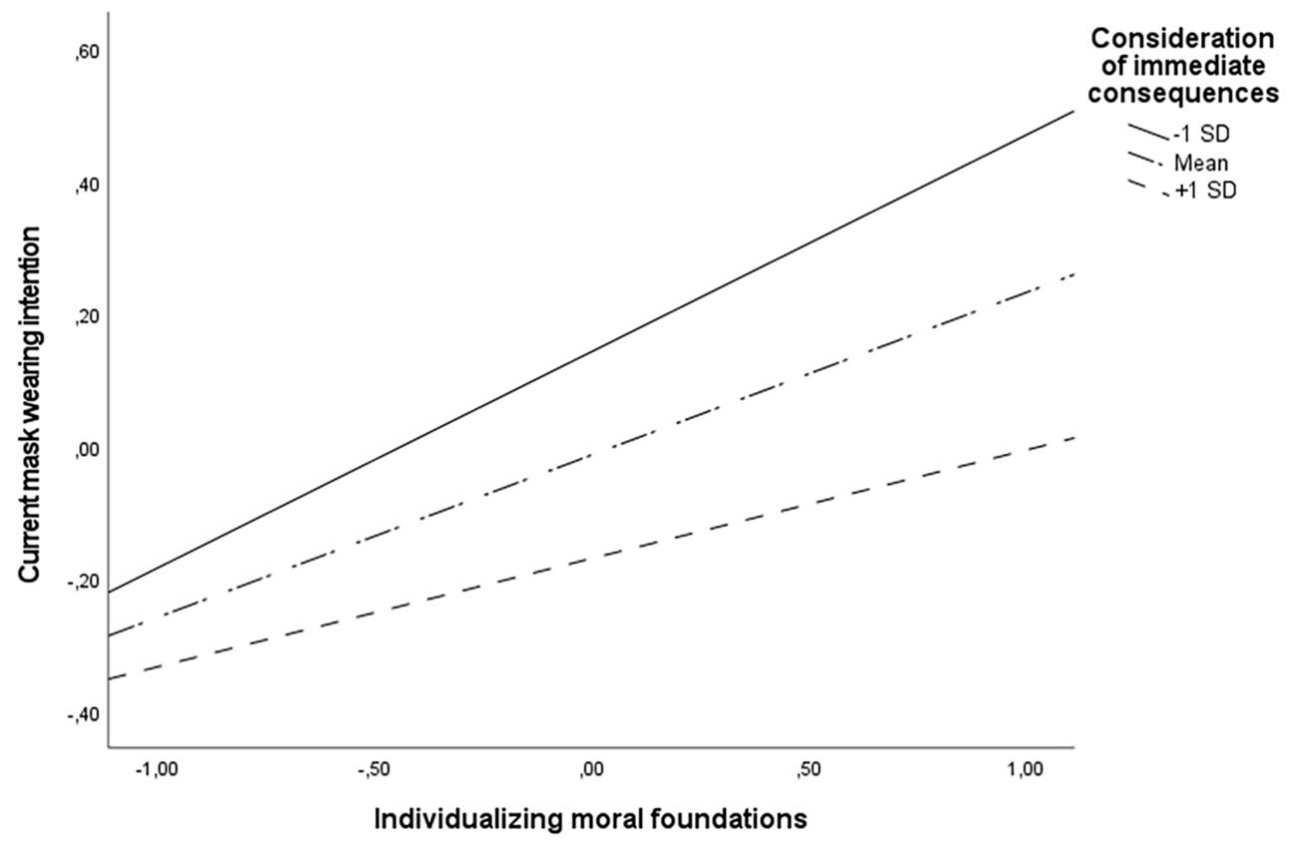
Interaction Between Individualizing Moral Foundations and Consideration of Immediate Consequences in Predicting Current Mask-Wearing Intention

Individualizing moral foundations were also related to current mask-wearing intentions on all levels of consideration of immediate consequences (other variables and interaction from the regression analysis controlled). However, the relationship differed in strength. It was the strongest when the consideration of immediate consequences was low, *B* = .33, *p* < .001; less strong when it was average, *B* = .25; *p* < .001, and the weakest when it was high, *B* = .16; *p* < .01. Figure 2 shows a visualization of this interaction.

Next, for exploratory purposes, a regression with interaction analysis was performed to predict mask-wearing refusal before the obligation revocation. Durbin-Watson's statistic for the whole model was 1.99. Table 4 shows the results of this analysis.

**Table 4:** Predicting Mask-Wearing Refusal Before the Revocation: Regression Analysis Results

Predictor	*B*	*SE*	β	*t*	*p*	VIF
1. Gender (0 = female, 1 = male)	14.49	3.61	.17	4.02	< .001	1.08
2. Individualizing moral foundations	-7.68	1.65	-.20	-4.66	< .001	1.08
3. Binding moral foundations	7.86	1.60	.20	4.91	< .001	1.06
4. Consideration of immediate consequences	3.02	1.53	.08	1.97	.049	1.01
5. Individualizing moral foundations * Binding moral foundations	1.67	1.64	.04	1.02	.307	1.07
6. Individualizing moral foundations * Consideration of immediate consequences	1.71	1.50	.05	1.14	.254	1.02
*F*(6; 538)	13.57					
*P*	< .001					
*R^2^_adj_*	.122					

Table 4 shows that the significant negative predictor of mask-wearing refusal before the obligation revocation were individualizing moral foundations. Positive and significant predictors were male gender, binding moral foundations, and consideration of immediate consequences. None of the interactions were significant.

## Discussion

The aim of the study was to test the role of moral foundations (individualizing versus binding) and consideration of immediate consequences (caring for the short-term gain of ceasing mask-wearing). In addition, it explored the interaction between individualizing and binding moral foundations as well as individualizing moral foundations and consideration of immediate consequences when it comes to predicting mask wearing intentions after the legal obligation to do so was revoked in Poland in March 2022. An additional model with the same predictors regarding the refusal to wear masks before the revocation was also tested.

The data supported all five hypotheses. For **H1**, individualizing moral foundations were consequently associated with mask-wearing intentions at all values of both moderators (the link differed in strength, which will be discussed below). This aligns with previous research stating that concern for care and justice is important for mask-wearing behaviors during COVID-19 (Chan, 2021). Valuing care means a person is concerned about others’ well-being (Clark et al., 2017). As masks were referred to in Poland as “a protection for others from your germs,” it could have been predicted that mainly other-oriented people would be more accepting of mask-wearing.

Regarding **H2**, binding moral foundations were negatively related to mask-wearing intentions after the revocation of the law. They were also related positively to the refusal to wear a mask when the obligation was present. This is congruent with how binding morality relates to preventive measure acceptance during the pandemic (Nowakowska & Zarazińska, 2023). It might be due to a willingness to preserve the *status quo* and avoid what is novel and what disturbs the known social order, which is characteristic of binding-oriented people (Graham et al., 2011). Another explanation is the linkage between binding moral foundations and lowered tolerance for ambiguity (Nowakowska & Zarazińska, 2023). Mask-wearing decisions bear some ambiguity: whether they are safe for individuals, whether the benefit of caring for others outweighs the concern for personal liberty, among others. Binding-oriented people may have decided not to comply to resolve this ambiguity and conflict. They might have preferred to remain with the familiar—that is, not using masks. Nevertheless, as tolerance of ambiguity was not measured in the current study, this interpretation is speculative and needs further research.

For **H3**, consideration of immediate consequences was negatively related to mask-wearing intention after the revocation. It was also positively related to a refusal to wear a mask when the obligation was in effect. People with a high consideration of immediate consequences take into account the results that are not distant in time (Strathman et al., 1994), for example, their comfort and lack of obligation to remember to put on a mask. That is why they might prefer the more straightforward solution of ignoring the obligation or giving up mask-wearing after the law was revoked.

Moreover, we observed interaction effects. For **H4** and **H5**, both binding moral foundations and consideration of immediate consequences moderated the role of individualizing moral foundations on mask-wearing intentions. The simple slope effect of individualizing moral foundations suggests that people who endorsed this type of morality—thus, caring for other people’s well-being (Graham et al., 2009)—generally tended to wear masks, and this effect is substantial. At the same time, binding foundations may incline toward following authorities and conforming to the group. Thus, for people who are individualizing and binding simultaneously, the concern for others might be related to a lower propensity to wear masks, presumably as a result of being fair to the authorities and ingroup. Consideration of immediate consequences might trigger a search for what feels more comfortable and what bears less immediately visible consequences (for example, negative judgments from others). That is why simultaneous individualizing morality and consideration of immediate consequences could be associated with lower mask-wearing intentions after revoking the law.

For the exploratory model, the moderating effects were not displayed in the relationship between individualizing moral foundations and mask-wearing before the revocation. This may be linked to the individualizing morality sense—if one is obliged to do something (as was the case when the mask-wearing mandate was in effect), it is a matter of fairness and reciprocity to comply. Other moral considerations or consequences should not be that important. Nevertheless, the data on mask-wearing during the pandemic is based on retrospective reports, and thus ought to be treated with caution.

Finally, the obtained results confirmed the previous findings regarding men’s lower tendency to wear protective masks (Chuang & Liu, 2020; Haischer et al., 2020; Looi, 2022; Palmer & Peterson, 2020). Summing up the differences between predictors for mask-wearing refusal before the revocation and intentions for mask-wearing after the revocation, women were less likely to refuse to wear a mask when the relevant law was in effect and more likely to continue wearing them after it was revoked. Binding moral foundations and consideration of immediate consequences were positively related to mask-wearing refusal before the revocation of the law and negatively to the intention to continue mask-wearing after the revocation. Individualizing moral foundations showed the opposite direction of correlations. Moreover, both binding moral foundations and consideration of immediate consequences suppressed the effect of individualizing moral foundations on further mask-wearing intentions.

Our study provides interesting insights for prevention and public health. First, it highlights the need for more communication and targeted public health messages aimed at men to enhance cooperation and responsibility beyond gender. Moreover, it shows that if health-related laws are in effect, individualizing and not binding moral foundations may play a role in complying with them. Presumably, to encourage binding-oriented people to comply with such laws, underlining the importance of particular behaviors to preserve group integrity, being loyal to other members of society, or referring to preserving purity or sanctity concerns could be helpful when planning health communications. Moreover, as people considering the immediate consequences are less likely to comply or sustain health-related behaviors, highlighting the short-term benefits of health-related behaviors can play a role. Nevertheless, wearing masks might be uncomfortable (thus having short-term negative consequences), which prevents engagement in such behavior. However, grounding the prevention messages in sound research results showing the short-term benefits for the community (e.g., fewer infected persons) or the individual (lack of contamination) can be helpful.

### Limitations and Future Research Directions

The study was unique in terms of its timing and allowed us to capture specific phenomena of high social and health relevance. However, it is not free of limitations. First, it was cross-sectional and allowed us to interpret the results only in the context at the time of data collection. Given that we collected the data right after the legal obligation to wear masks in public places was revoked, we were able to capture the behavioral intentions right when the relevant circumstances changed. However, due to this study’s timing, the data on mask-wearing during the pandemic was only retrospective, and the model predicted it might be prone to bias related to recalling one’s own behavior from the past.

Given that a very short time passed after the revocation of the abovementioned laws, we cannot infer from our data how the investigated individual differences related to sustained mask-wearing behaviors after the pandemic. Moreover, the study was done during the first months of the war in Ukraine, which was a period of high activity in Polish society for helping refugees (Byrska, 2023). The attention of Polish citizens could have shifted from the pandemic and related restrictions to the armed conflict taking place in the neighboring country and the refugee crisis.

We need to acknowledge that the newest scientific reviews question the relevance of mask-wearing in preventing the spread of COVID-19 (Jefferson et al., 2023). However, as mask-wearing was considered socially responsible at the time of the study, our study contributes to understanding people’s intentions to continue expected health behaviors in the aftermath of a pandemic.

Moreover, all the declarations were based on self-report, which is somewhat dependent on the need for social approval. There was also a gender imbalance typical for snowball sampling studies conducted on social media. The online design and the data collection method, which did not involve context-setting or the presence of a researcher, did not allow control over the participants’ attentiveness while they answered the questions. The level of engagement of respondents is unknown, and as in many online studies, there is a risk that some of them did not take the study seriously and were filling out the survey without consideration.

Future studies could explore the topic of socially responsible behaviors in the framework of moral foundations theory to find out whether the effect is similar to other behaviors and intentions. More research and practical interventions are also needed to tailor public health communication to men in order to enhance their compliance with COVID-19 and other disease preventive measures.

## Supplementary Materials

For this article, the following Supplementary Materials are available:

- Data. (Nowakowska, 2024)
- Codebook. (Nowakowska, 2024)
- Materials. (Nowakowska, 2024)

## Data Availability

For this article, data, codebook and materials are available at Nowakowska (2024).

## Appendix

### Factor Analysis for the Mask Behavior Survey

The method used: Principal Component Analysis, no rotation. Criterion for number of factors chosen: Eigenvalue > 1.

**Table:**

Questionnaire Item	Factor loading
Since the revocation of the obligation to wear protective masks, I do not wear one in places where it is not required (R).	.789
Despite the revocation of the obligation to wear protective masks, I still wear one in places where it is not required.	.858
If someone asks me to wear a protective mask and it is not required in that place, I will wear one.	.864
If someone asks me to wear a protective mask and it is not required in that place, I will refuse to do so (R).	.852
If during a 1:1 conversation, I would be without a protective mask and another person would wear one, I would wear one too.	.875
If, during a meeting with several people, I would be without a protective mask and the majority would wear masks, I would wear one too.	.888

*Note*. R–reverse-coded item.

One factor was revealed, explaining 73.09% of the total variance; K-M-O metric = .715; Bartlett’s sphericity test χ*^2^*(15) = 3403.48; *p* < .001, Cronbach’s α = .93.

## References

[r1] Boulos, L., Curran, J. A., Gallant, A., Wong, H., Johnson, C., Delahunty-Pike, A., Saxinger, L., Chu, D., Comeau, J., Flynn, T., Clegg, J., & Dye, C. (2023). Effectiveness of face masks for reducing transmission of SARS-CoV-2: A rapid systematic review. Philosophical Transactions of the Royal Society A, 381(2257), 20230133. 10.1098/rsta.2023.0133 37611625 PMC10446908

[r2] Byrska, O. (2023). Civil crisis management in Poland: The first weeks of the relief in Russian war on Ukraine. Journal of Genocide Research, 25(3–4), 463–470. 10.1080/14623528.2022.2079196

[r3] Chan, E. Y. (2021). Moral foundations underlying behavioral compliance during the COVID-19 pandemic. Personality and Individual Differences, 171, 110463. 10.1016/j.paid.2020.110463 33106715 PMC7577686

[r4] Chi, X., Han, H., & Kim, S. (2022). Protecting yourself and others: Festival tourists’ pro-social intentions for wearing a mask, maintaining social distancing, and practicing sanitary/hygiene actions. Journal of Sustainable Tourism, 30(8), 1915–1936. 10.1080/09669582.2021.1966017

[r5] Chuang, Y., & Liu, J. C. E. (2020). Who wears a mask? Gender differences in risk behaviors in the COVID-19 early days in Taiwan. Economic Bulletin, 40(4), 2619–2627. https://EconPapers.repec.org/RePEc:ebl:ecbull:eb-20-00882

[r6] Clark, C. B., Swails, J. A., Pontinen, H. M., Bowerman, S. E., Kriz, K. A., & Hendricks, P. S. (2017). A behavioral economic assessment of individualizing versus binding moral foundations. Personality and Individual Differences, 112, 49–54. 10.1016/j.paid.2017.02.052

[r7] Czarnecki, Ł., & Skowrońska, M. (2022). The unlawful and unequal wearing of masks: The case of Poland during COVID-19. In N. Suzuki, X. Mellet, S. Annaka & M. Endo (Eds.), *Public behavioural responses to policy making during the pandemic* (pp. 105–115). Routledge. 10.4324/9781003244127

[r8] Day, M. V., Fiske, S. T., Downing, E. L., & Trail, T. E. (2014). Shifting liberal and conservative attitudes using moral foundations theory. Personality and Social Psychology Bulletin, 40(12), 1559–1573. 10.1177/0146167214551152 25286912 PMC4858184

[r9] Frydrysiak, A. A. (2018). *Polska adaptacja i walidacja zrewidowanej Skali rozważania przyszłych konsekwencji* [Polish adaptation and validation of the revised Consideration of Future Consequences scale] [Unpublished thesis]. Adam Mickiewicz University.

[r10] Gostin, L. O., & Hodge, J. G. (2020). US emergency legal responses to novel coronavirus: Balancing public health and civil liberties. Journal of the American Medical Association, 323(12), 1131–1132. 10.1001/jama.2020.2025 32207808

[r11] Graham, A., Cullen, F. T., Pickett, J. T., Jonson, C. L., Haner, M., & Sloan, M. M. (2020). Faith in Trump, moral foundations, and social distancing defiance during the coronavirus pandemic. Socius: Sociological Research for a Dynamic World, 6. 10.1177/2378023120956815

[r12] Graham, J., Haidt, J., & Nosek, B. A. (2009). Liberals and conservatives rely on different sets of moral foundations. Journal of Personality and Social Psychology, 96(5), 1029–1046. 10.1037/a0015141 19379034

[r13] Graham, J., Nosek, B. A., Haidt, J., Iyer, R., Koleva, S., & Ditto, P. H. (2011). Mapping the moral domain. Journal of Personality and Social Psychology, 101(2), 366–385. 10.1037/a0021847 21244182 PMC3116962

[r14] Haidt, J., Graham, J., & Joseph, C. (2009). Above and below left–right: Ideological narratives and moral foundations. Psychological Inquiry, 20(2–3), 110–119. 10.1080/10478400903028573

[r15] Haidt, J. (2012). *The righteous mind: Why good people are divided by politics and religion*. Pantheon Books.

[r16] Haischer, M. H., Beilfuss, R., Hart, M. R., Opielinski, L., Wrucke, D., Zirgaitis, G., Uhrich, T. D., & Hunter, S. K. (2020). Who is wearing a mask? Gender-, age-, and location-related differences during the COVID-19 pandemic. PLoS One, 15(10), e0240785. 10.1371/journal.pone.0240785 33057375 PMC7561164

[r17] Hayes, A. F. (2018). *Introduction to mediation, moderation, and conditional process analysis: A regression-based approach*. Guilford Publications.

[r18] Howard, J., Huang, A., Li, Z., Tufekci, Z., Zdimal, V., Van Der Westhuizen, H. M., von Delft, A., Price, A., Fridman, L., Tang, L.-H., Tang, V., Watson, G. L., Bax, C. E., Shaikh, R., Questier, F., Hernandez, D., Chu, L. F., Ramirez, C. M., & Rimoin, A. W. (2021). An evidence review of face masks against COVID-19. Proceedings of the National Academy of Sciences of the United States of America, 118(4), e2014564118. 10.1073/pnas.2014564118 33431650 PMC7848583

[r19] Janoff-Bulman, R., & Carnes, N. C. (2013). Surveying the moral landscape: Moral motives and group-based moralities. Personality and Social Psychology Review, 17(3), 219–236. 10.1177/1088868313480274 23504824

[r20] Jarmakowski-Kostrzanowski, T., & Jarmakowska-Kostrzanowska, L. (2016). Polska adaptacja Kwestionariusza kodów moralnych (MFQ-PL) [Polish adaptation of the Moral Foundations Questionnaire (MFQ-PL)]. Psychologia Spoleczna, 39, 489–508. 10.7366/1896180020163908

[r21] Jefferson, T., Dooley, L., Ferroni, E., Al-Ansary, L. A., van Driel, M. L., Bawazeer, G. A., Jones, M. A., Hoffmann, T. C., Clark, J., Beller, E. M., Glasziou, P. P., & Conly, J. M. (2023). Physical interventions to interrupt or reduce the spread of respiratory viruses. Cochrane Database of Systematic Reviews, 1, CD006207. 10.1002/14651858.CD006207.pub6 36715243 PMC9885521

[r22] Joireman, J., Balliet, D., Sprott, D., Spangenberg, E., & Schultz, J. (2008). Consideration of future consequences, ego-depletion, and self-control: Support for distinguishing between CFC-Immediate and CFC-Future sub-scales. Personality and Individual Differences, 45(1), 15–21. 10.1016/j.paid.2008.02.011

[r23] Joireman, J., Shaffer, M. J., Balliet, D., & Strathman, A. (2012). Promotion orientation explains why future-oriented people exercise and eat healthy: Evidence from the two-factor Consideration of Future Consequences-14 Scale. Personality and Social Psychology Bulletin, 38(10), 1272–1287. 10.1177/0146167212449362 22833533

[r24] Kaul, V., & Palmer, Z. D. (2023). “You are responsible for your own safety”: An intersectional analysis of mask-wearing during the COVID-19 pandemic. Social Currents, 10(4), 363–380. 10.1177/23294965221145904

[r25] Kaźmierczak, I., Jelonkiewicz-Sterianos, I., Nowakowska, I., Rajchert, J., Zajenkowska, A., Jakubowska, A., & Bodecka-Zych, M. (2022). Does pandemic denial help or harm? Belief in COVID-19 and experience of it affect psychological consequences of using neurotic defence mechanisms. Psychiatria i Psychologia Kliniczna, 22(3), 151–157. 10.15557/PiPK.2022.0020

[r26] Kupers, T. A. (2005). Toxic masculinity as a barrier to mental health treatment in prison. Journal of Clinical Psychology, 61(6), 713–724. 10.1002/jclp.20105 15732090

[r27] Lang, J., Erickson, W. W., & Jing-Schmidt, Z. (2021). #MaskOn! #MaskOff! Digital polarization of mask-wearing in the United States during COVID-19. PLoS One, 16(4), e0250817. 10.1371/journal.pone.0250817 33909669 PMC8081244

[r28] Looi, K. H. (2022). Contextual factors of face mask wearing during the COVID-19 pandemic and recommendations to increase compliance rate. Malaysian Journal of Medical Sciences : MJMS, 29(1), 147–153. 10.21315/mjms2022.29.1.15 35283678 PMC8887986

[r29] Mejova, Y., Kalimeri, K., & Morales, G. D. F. (2023). Authority without care: Moral values behind the mask mandate response. *Proceedings of the International AAAI Conference on Web and Social Media, 17,* 614–625. 10.1609/icwsm.v17i1.22173

[r30] Mendez-Brito, A., El Bcheraoui, C., & Pozo-Martin, F. (2021). Systematic review of empirical studies comparing the effectiveness of non-pharmaceutical interventions against COVID-19. Journal of Infection, 83(3), 281–293. 10.1016/j.jinf.2021.06.018 34161818 PMC8214911

[r31] Milesi, P., & Alberici, A. I. (2018). Pluralistic morality and collective action: The role of moral foundations. Group Processes & Intergroup Relations, 21(2), 235–256. 10.1177/1368430216675707

[r33] Nowakowska, I. (2024). *Mask-wearing study 2022* [OSF project page containing code, data, and study materials]. OSF. 10.17605/OSF.IO/Q5JBC

[r32] Nowakowska, I., & Zarazińska, A. (2023). What is important for you makes you think about the pandemic differently: Moral foundations, pandemic-related fears and convictions. Studies in Psychology, 44(2–3), 282–320. 10.1080/02109395.2023.2207300

[r34] Palmer, C. L., & Peterson, R. D. (2020). Toxic mask-ulinity: The link between masculine toughness and affective reactions to mask wearing in the COVID-19 era. Politics & Gender, 16(4), 1044–1051. 10.1017/S1743923X20000422

[r35] Schwartz, S. H. (1970). Moral decision making and behavior. In M. Macauley & L. Berkowitz (Eds.), *Altruism and helping behavior* (pp. 127–141). Academic Press.

[r36] Strathman, A., Gleicher, F., Boninger, D. S., & Edwards, C. S. (1994). The consideration of future consequences: Weighing immediate and distant outcomes of behavior. Journal of Personality and Social Psychology, 66(4), 742–752. 10.1037/0022-3514.66.4.742

[r37] Talic, S., Shah, S., Wild, H., Gasevic, D., Maharaj, A., Ademi, Z., Li, X., Xu, W., Mesa-Eguiagaray, I., Rostron, J., Theodoratou, E., Zhang, X., Motee, A., Liew, D., & Ilic, D. (2021). Effectiveness of public health measures in reducing the incidence of Covid-19, SARS-CoV-2 transmission, and Covid-19 mortality: Systematic review and meta-analysis. BMJ (Clinical Research Ed.), 375, e068302. 10.1136/bmj-2021-068302 34789505 PMC9423125

[r38] Thunstrӧm , L., Newbold, S. C., Finnoff, D., Ashworth, M., & Shogren, J. F. (2020). The benefits and costs of using social distancing to flatten the curve for COVID-19. Journal of Benefit-Cost Analysis, 11(2), 179–195. 10.1017/bca.2020.12

[r39] Waling, A. (2019). Problematising ‘toxic’ and ‘healthy’ masculinity for addressing gender inequalities. Australian Feminist Studies, 34(101), 362–375. 10.1080/08164649.2019.1679021

[r40] Waytz, A., Iyer, R., Young, L., Haidt, J., & Graham, J. (2019). Ideological differences in the expanse of the moral circle. Nature Communications, 10, 4389. 10.1038/s41467-019-12227-0 31558713 PMC6763434

